# Cloning of Bovine herpesvirus type 1 and type 5 as infectious bacterial artifical chromosomes

**DOI:** 10.1186/1756-0500-2-209

**Published:** 2009-10-14

**Authors:** Evgeni Gabev, Cornel Fraefel, Mathias Ackermann, Kurt Tobler

**Affiliations:** 1 Institute of Virology, University of Zurich, Switzerland

## Abstract

**Background:**

Bovine herpesviruses type 1 (BoHV1) and type 5 (BoHV5) are two closely related pathogens of cattle. The identity of the two viruses on the amino acid level averages 82%. Despite their high antigenetic similarities the two pathogens induce distinctive clinical signs. BoHV1 causes respiratory and genital tract infections while BoHV5 leads to severe encephalitis in calves.

**Findings:**

The viral genomes of BoHV1 and BoHV5 were cloned as infectious bacterial artificial chromosomes (BACs). First, recombinant viruses carrying the genetic elements for propagation in bacteria were generated. Second, DNA from these recombinant viruses were transferred into prokaryotic cells. Third, DNA from these bacteria were transferred into eukaryotic cells. Progeny viruses from BAC transfections showed similar kinetics as their corresponding wild types.

**Conclusion:**

The two viral genomes of BoHV1 and BoHV5 cloned as BACs are accessible to the tools of bacterial genetics. The ability to easily manipulate the viral genomes on a molecular level in future experiments will lead to a better understanding of the difference in pathogenesis induced by these two closely related bovine herpesviruses.

## Findings

### Background and aim of this study

Bovine herpesviruses type 1 (BoHV1) and type 5 (BoHV5) belong to the subfamily *Alphaherpesvirinae *and are closely related pathogens of cattle, with coding capacity of more than seventy open reading frames (ORF's) [1]. Despite their high identity (82%) on the amino acid (aa) level leading to a similar antigenetic repertoire [2], these two viruses induce distinctive clinical signs. BoHV1 causes respiratory and genital tract symptoms including infectious rhinotracheitis, pustular vulvovaginitis, and abortion [3]. BoHV5 causes severe encephalitis in calves and experimentally in rabbits and mice [4-7]. In order to have an approach to elucidate the differences in the pathogenesis of these two viruses on a molecular level, we have set out to clone the entire genomes of BoHV1 (strain Jura) and BoHV5 (strain N569) as bacterial artificial chromosomes (BACs). As such, these cloned genomes will become accessible to the tools of bacterial genetics [8], allowing facilitated generation of recombinant viruses in future experiments. In fact, BoHV1 genomes of three different strains were previously cloned as BACs by Mahony *et al. *(strain V155) [9], Trapp *et al. *(strain Schönböken) [10], and Liu *et al. *(strain Cooper) [11]. However, in contrast to the approach reported by Mahony *et al. *and Trapp *et al.*, we introduced the heterologous sequences flanked by *loxP *sites into a intergenic region of BoHV1 genome. The benefit of this strategy is given by the fact that none of the viral DNA coding sequences are disrupted. The heterologous sequences can be excised on demand by *Cre *recombinase, which depicts another advantage over the previously used methods. Unlike the construct of Mahoney *et al. *[9] our BoHV1 BAC clone harbours GFP coding sequence as part of the heterologous sequences, which easily enables the monitoring of virus plaque formation using fluorescent microscopy. The BoHV1 BAC reported by Liu *et al. *[11] has similar genetic features as our BoHV1 BAC though we cloned the strain we investigated most in our lab and we report the cloning of BoHV5 as BAC, which is not reported yet.

The cloning of BoHV genomes as BACs can be divided into three steps: First, genetic elements required for DNA replication and selection in bacteria were inserted by homologous recombination into the virus genomes. Second, circular viral DNA was extracted from infected cells and transferred into bacteria. Third, viruses were reconstituted upon transfection of BAC-DNA into eukaryotic cells.

### Generation of recombinant viruses in eukaryotic cells

**Figure 1:**
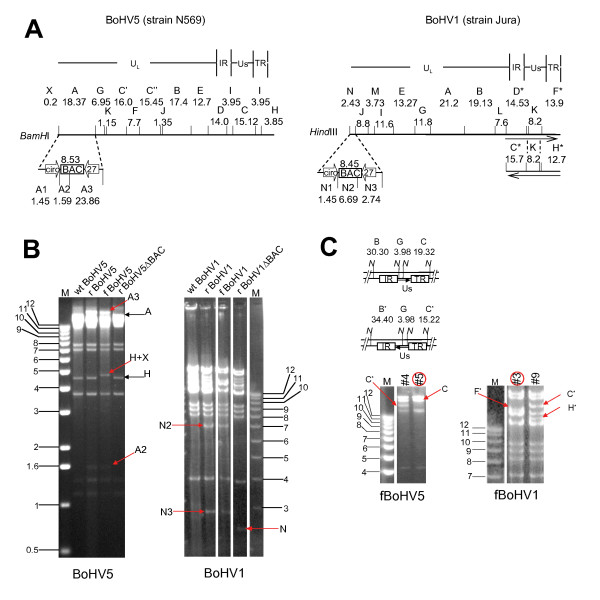
**Strategy for cloning the full-length genomes of wt BoHV5 and wt BoHV1 as BACs and restriction enzyme analyses (REA). Panel A**: Restriction maps of BoHV5 (*Bam*HI; left) and BoHV1 (*Hin*dIII; right). Division in unique long (U_L_), unique short (U_S_), internal repeat (IR) and terminal repeat (TR) is shown on top, fragments are lettered alphabetically and sizes are indicated in kbp. Left ends of genomes are additionally blown up to map the site of BAC backbone insertion between CIRC (circ) and BICP27 (27). Upon insertion into BoHV5, *Bam*HI fragment A is replaced by fragments A1, A2, and A3. Likewise, insertion into BoHV1, *Hin*dIII fragment N is replaced by fragments N1, N2 and N3. **Panel B**: Restriction enzyme digests of wt BoHV, rBoHV, fBoHV and rBoHVΔBAC. Fragments of interest are labelled as in panel A: **Panel C**: Possible two orientations of U_S _within fBoHV5 and fBoHV1 respectively. *Hin*dIII map of both orientations for BoHV1 is part of panel A, *Nde*I of both orientations for BoHV5 is given above the REA. fBoHV5 BAC #5 and fBoHV1 BAC #3 pointed with red circles were used for further experiments.

To generate recombinant (r) BoHV viruses carrying the BAC cassette, eukaryotic cells were cotransfected with viral DNA of BoHV1 or BoHV5 with appropriate plasmids, which allow the insertion of heterologous elements into the viral genomes over homologous recombination. The viral DNAs used for cotransfections were isolated from sucrose cushion (12 ml 35% in TNE) purified virions (collected from one infected 150 cm^2 ^tissue culture flask) by SDS (1%) and Proteinase K (0.6 g/l) treatment and phenol/chloroform extraction. In order to construct the plasmid used for cotransfections with BoHV1 DNA (pBS-Belo-BoHV1), sequence elements necessary for replication and selection of DNA in bacteria and eGFP under the control of the Cytomegalovirus Immediate Early (CMV-IE) promoter as a reporter protein were flanked by two *loxP *sites and on each side a stretch of BoHV1 specific DNA sequences allowing homologous recombination with viral DNA [12,13]. Two *Nsi*I sites just outside the *lox*P sites were introduced into the plasmid pBS-Belo-BoHV1 and its sequence was deposited in GenBank (accession number AY665170). These *Nsi*I sites were used for exchange of the BoHV1 specific sequences with the BoHV5 specific sequences resulting in the plasmid pBS-Belo-BoHV5. Recombinant (r) BoHV1, carrying the BAC cassette, was generated in Madine Darby Bovine Kidney cells (MDBK cells) (50% confluent on 60 mm plates) cotransfected with 4.5 μg BoHV1 strain Jura DNA and 1 μg pBS-Belo-BoHV1, and 0.5 μg of plasmid pBCMV26 by BES buffered CaCl_2 _precipitation method [14]. Likewise, rBoHV5 was generated in Vero 2-2 cells (1.2 × 10^6 ^on 60 mm plate) cotransfected with 2 μg BoHV5 strain N569 DNA and 0.2 μg pBS-pBelo-BoHV5 and 0.2 μg of plasmid pBCMV26 by Lipofectamin (Invitrogen) [15]. The recombination cassette was targeted to a noncoding region of the viruses, which is located between the UL54 ORF (encoding BICP27) and the neighboring ORF encoding the circ protein (Figure 1A). Plasmid pBCMV26 [16] encoding for the Bovine infected cell protein 0 (*BICP0*) gene under the control CMV-IE was included in the cotransfection to increase the infectivity of transfected BoHV1 DNA (Fraefel, unpublished results). Both, rBoHV1 and rBoHV5 carrying BAC cassettes and expressing the enhanced green fluorescent protein were plaque purified in three consecutive rounds on MDBK cells under DMEM supplemented with 0.75% agarose.

### Transfer and propagation of viral DNA in bacteria

A typical feature of herpesvirus genomes is their circular conformation early in infection [17]. Since circularity of DNA is a prerequisite for the propagation of plasmids in bacteria, such DNA was isolated from infected cells. In brief, confluent MDBK cells grown in 60 mm plates were infected at a multiplicity of infection (moi) of 5 with rBoHV1 or rBoHV5. Two (rBoHV1) or four (rBoHV5) hours post infection (hpi) the monolayer was washed once with PBS, then rinsed using 10 mM EDTA, 10 mM Tris-HCI, pH 8.0 buffer, subsequently the cells were scraped from the tissue culture dish into 0.7 ml of the same buffer. Proteinase K and SDS were added to final concentrations of 0.25 g/l and 0.6%, respectively. Three microliters of circular viral DNA were taken to electroporate *E. coli *(ElectroMAX™ DH10B™, Invitrogen) using 0.1 cm-cuvettes in a BioRad Gene Pulser (1.8 kV voltage, 200 Ω impedance, 25 μF capacity). The electroporated bacteria were grown on Luria-Bertani (LB)-plates supplemented with 12.5 μg/ml chloramphenicol. Colonies were picked and expanded in liquid LB medium supplemented with 12.5 μg/ml chloramphenicol.

DNA was extracted from bacterial cultures and analysed by restriction enzyme analysis. The resulting BACs were designated fBoHV5 BAC and fBoHV1 BAC, respectively. The restriction enzyme patterns confirmed the integrity of the viral DNA and the insertion of the replicon sequence at the desired location (Figure 1A to 1B). Of note, the unique short segment (U_S_) of the genome did not invert relative to the long segment (U_L_) in the BAC DNA. The herpes-genomic DNA as part of the BACs were frozen in the following isomeric arrangement: U_L _(circ through BICP0), internal repeat, U_S _(US1.67 through US9), terminal repeat (Figure 1C).

### Viral growth curve analysis

**Figure 2:**
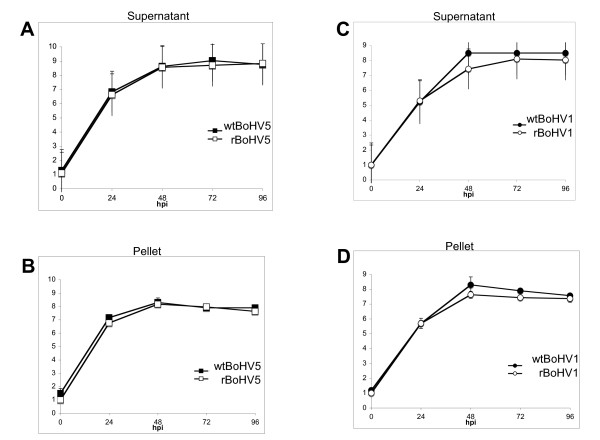
**Growth kinetics of rBoHV5 and rBoHV1 mutants versus wt BoHV5 and wt BoHV1**. MDBK cells were infected at moi of 0.01 PFU with different viruses and harvested at various times post inoculation as indicated. The virus yields at each time point were determined by titration. **Panels A and B**: wt BoHV5 (full squares) and rBoHV5 (open squares). **Panels C and D**: wt BoHV1 (full circles) and rBoHV1 (open circles). Cell-free (supernatant, panels A and C) and cell-associated virus (pellets, panels B and D) were titrated separately. The x-axis represents the time scale post infection. Virus titers (y-axis) are expressed as TCID_50_/ml.

Furthermore, we wanted to address the question whether the insertion of 8.5 kb heterologous DNA sequence in BoHV genomes influences viral replication *in vitro*? To answer this question viral growth curve analyses were performed. MDBK cells were infected with respective viruses at multiplicity of infection (moi) of 0.01. After 2 h at 4°C, the temperature was shifted for 1 h to 37°C to allow virus penetration. The inoculum was removed, the cells were washed twice with PBS and overlaid with fresh DMEM. Immediately thereafter (time 0), and after 24, 48, 72, and 96 h of incubation at 37°C, the infected cells were scraped into the cell culture medium and clarified by centrifugation (311 × g for 10 min). The supernatant was removed and analyzed separately, the cell pellet was resuspended in fresh DMEM. Infectious virus was harvested following three cycles of freezing/thawing and low speed centrifugation to remove cell debris. The amount of infectious virus in the supernatants as well as in the pellets was determined by separate titration assays in 96 well plates. Each titration was performed in three independent experiments and viral titers were determined as TCID_50_/ml in MDBK cells (Figure 2).

Briefly, the growth curves and the final titers were similar for all viruses, although wt BoHV5 and rBoHV5 reached generally, though not significantly, higher titers than wt BoHV1 and rBoHV1 mutant. Furthermore, the amounts of cell-free virus slightly exceeded the amount of cell-associated virus. However, the viruses with retained BAC cassette had similar kinetics to the respective wt BoHV viruses.

### Deletion of BAC cassettes in rBoHV's by *cre *mediated recombination

MDBK cells were cotransfected with rBoHV1 or rBoHV5 viral DNA and a *Cre *recombinase expressing vector p116.006 [18]. Three days post transfection five viral plaques were randomly collected and plaque purified three times as described above. Finally, viral DNA from non-eGFP fluorescent progeny was extracted and characterized by restriction enzyme analysis in order to verify the deletion of the BAC. Upon reconstitution of BAC DNA, Unique short (U_S_) segment does again invert relative to Unique long (U_L_) segment (Figure 1B).

**Figure 3:**
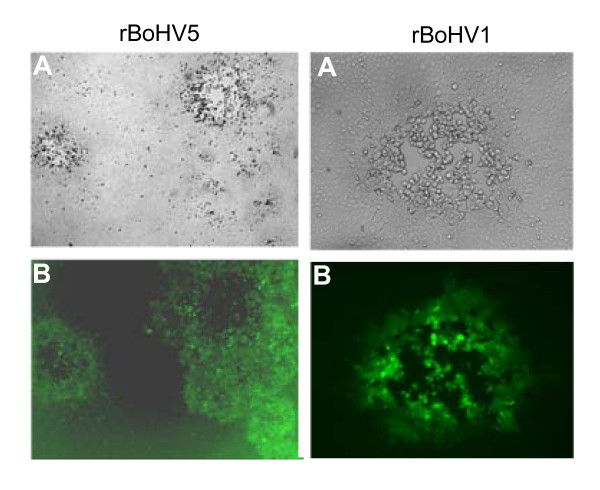
**Recombinant viruses carrying the BAC-backbone and eGFP expression cassette**. MDBK cells were infected with BAC derived rBoHV5 (panels A and B) or rBoHV1 (panels C and D). Panels A and C are captured under normal light. Panels B and D are GFP-filtered micrographs.

In conclusion, our results describe the successful cloning of both, the BoHV5 and BoHV1 genome as BACs. Upon transfection of MDBK cells with BoHV5 or BoHV1 BAC DNA green fluorescent plaques were generated (Figure 3). The BAC cassettes were successfully deleted upon co-transfection of MDBK cells with BoHV5 BAC DNA or BoHV1 BAC DNA and *Cre *recombinase expressing vector, resulting in black viral plaques formation. The viral replication kinetics of BAC derived viruses was not altered through insertion of 8.5 kb heterologous DNA as they replicated similar to their respective wild types. Indeed, preliminary experiments indicate (data not shown) that the rescued viruses did retain their virulence, at least in a previously described mouse model [4]. Thus, the overall properties of wt and rescued viruses are well preserved in the BAC. This is important since BoHV5 isolates may attenuate rapidly upon passaging in cell cultures. In contrast, BoHV1 and BoHV5 genomes frozen in BACs remain very stable.

Thus, these cloned genomes will be useful tools towards elucidating the differences in the pathogenesis of BoHV1 and BoHV5, using bacterial genetics for genome manipulation [8].

## Competing interests

The authors declare that they have no competing interests.

## Authors' contributions

EG constructed the transfer plasmid pBS-pBelo-BHV5, generated fBoHV5 and rBoHV5ΔBAC, reconstituted virus from fBoHV5 DNA, and performed the viral growth curve analyses. CF generated rBoHV5. KT constructed the transfer plasmid pBS-pBelo-BoHV1 and p116.006, generated rBoHV1, fBoHV1, rBoHV1ΔBAC, and reconstituted virus from fBoHV1 DNA. MA made substantial intellectual contributions. All authors read and approved the manuscript.
